# The Immune System, Cytokines, and Biomarkers in Autism Spectrum Disorder

**DOI:** 10.1007/s12264-017-0103-8

**Published:** 2017-02-25

**Authors:** Anne Masi, Nicholas Glozier, Russell Dale, Adam J. Guastella

**Affiliations:** 1 Autism Clinic for Translational Research, Brain and Mind Centre, Central Clinical School, Sydney Medical School, University of Sydney, Sydney, New South Wales 2050 Australia; 2 Childrens Hospital at Westmead Clinical School, University of Sydney, Sydney, New South Wales 2145 Australia

**Keywords:** Autism Spectrum Disorder, Cytokine, Immune system

## Abstract

Autism Spectrum Disorder (ASD) is a pervasive neurodevelopmental condition characterized by variable impairments in communication and social interaction as well as restricted interests and repetitive behaviors. Heterogeneity of presentation is a hallmark. Investigations of immune system problems in ASD, including aberrations in cytokine profiles and signaling, have been increasing in recent times and are the subject of ongoing interest. With the aim of establishing whether cytokines have utility as potential biomarkers that may define a subgroup of ASD, or function as an objective measure of response to treatment, this review summarizes the role of the immune system, discusses the relationship between the immune system, the brain, and behavior, and presents previously-identified immune system abnormalities in ASD, specifically addressing the role of cytokines in these aberrations. The roles and identification of biomarkers are also addressed, particularly with respect to cytokine profiles in ASD.

## Introduction

Autism Spectrum Disorders (ASDs) are complex, pervasive neurodevelopmental conditions with a largely unknown etiology and a significant male bias. ASDs are behaviorally defined and characterized by deficits in social communication and interaction, and the presence of restricted, repetitive patterns of behavior, interests, or activities [1]. Developmental trajectories and patterns of severity vary substantially, with all facets of daily functioning potentially impacted. The vast clinical heterogeneity is a hallmark. Comorbid psychiatric and medical conditions are frequently reported, including social anxiety disorder, attention deficit disorder, immune system abnormalities, gastrointestinal disorders, mitochondrial dysfunction, sleep disorders, and epilepsy [2–6]. The multifaceted nature of the condition has resulted in investigations aiming to characterize biological subtypes of ASD. However, an understanding of the biological mechanisms driving pathophysiology is evolving. Immune system aberrations, including altered cytokine profiles, are believed to have a role in ASD [7, 8]. Accumulating evidence of alterations in central and peripheral immune system functioning supports the proposal that there is a subgroup of individuals with ASD who have some form of immune system dysregulation [9]. Altered cytokine levels may facilitate the identification of ASD subtypes as well as provide biological markers of the response to effective treatments.

## The Immune System

The immune system is a complicated group of defense mechanisms that are triggered in order to protect an organism from disease- or illness-causing pathogens, which include bacteria, viruses, fungi, and parasites. An antigen, considered foreign by the host’s body, is a molecule that stimulates the immune system to produce antibodies which function to identify and neutralize or remove the antigen. Many interconnected organs, molecules, cells, and pathways play roles in a fully functional immune system, which is comprised of two interconnected systems of immunity: innate and adaptive [10]. These two systems work together to protect the body from pathogens.

Innate immunity refers to first-line defense mechanisms that respond to an infection immediately or within hours of the host being attacked by a pathogen. The innate immune response relies on physical barriers, such as the epithelial layers of the skin and mucosal and glandular tissue surfaces connected to the body’s openings, as well as chemical barriers, which include soluble antimicrobial proteins and peptides, and an acidic pH. When pathogens breach these barriers, cellular innate immune responses are triggered through a pathogen-recognition process involving an array of cells with cell surface and intracellular receptors. Groups of pathogens present with characteristic pathogen-associated molecular patterns, which are recognized by pattern recognition receptors expressed by many different immune cells [11]. Some cells are activated to phagocytose and degrade the pathogen, a process through which macrophages and neutrophils engulf and destroy extracellular microbes. Cell receptors can also be activated, causing the cells to produce antimicrobial substances that eliminate the pathogens. Other cellular activation processes lead to the production of cytokines and chemokines, which are proteins that recruit cells, molecules, and fluid to sites of infection. This procedure results in the physiological changes known as inflammation [10]. Other effectors of innate immunity are natural killer (NK) cells that, through cytokine production or cytotoxicity, contain infections until an adaptive immune response is initiated.

When innate immunity is insufficient, for example when features of certain pathogens allow them to evade the defense mechanisms of the innate immune system, the adaptive immune response is activated. The adaptive response is initiated a few days after initial exposure to the pathogen and breach of the physical or chemical barriers. This response is more comprehensive and antigen-specific than the innate response. The adaptive immune system is able to recognize, eliminate, and remember pathogens. The innate immune system is not considered to have this memory component. However, demonstrations of NK cell memory in viral infections indicate that these cells have attributes of both innate and adaptive immunity [12]. There are two types of adaptive immune response, both carried out by lymphocytes. The first type of response is an antibody response carried out by B lymphocytes, or B cells, which, when activated by an antigen, secrete antibodies, also known as immunoglobulins. B cells are formed in the bone marrow. The second type of adaptive immune response is a cell-mediated immune response where activated T cells specifically recognize and neutralize or eliminate antigens. T cells mature in the thymus, which is a lymphoid organ of the immune system. Residual B and T cells remaining after antigen exposure function as memory cells that are activated by subsequent pathogen challenges.

The innate and adaptive immune responses discussed above are characteristic of a regulated immune system contributing to the maintenance of homeostasis and preventing disruptions of normal functions of the body. A regulated immune system requires an optimal balance of pro- and anti-inflammatory signaling. Inflammation may not be a problem in isolation, but if an unregulated or dysregulated immune system responds to physiological changes initiated by pathogens, then the inflammation may be problematic. An aberrant immune system may manifest as upregulation of the inflammatory/immune response or as immune deficiency compromising host defense. Allergies, asthma, and autoimmune disorders are all conditions associated with immune dysfunction.

## Cytokines and the Immune System

Cytokines are cell-signaling molecules that facilitate communication among cells of both the innate and adaptive immune systems. They are primary regulators of inflammation, coordinating the response to infection and associated immune challenges and are involved in a multitude of biological processes. As part of an integrated network, cytokines stimulate and modulate immune system activity and induce their own synthesis and the synthesis of other cytokines. They are typically soluble molecules although some remain cell-bound. Cytokines can be broadly classified into three groups based on the type of immune response: adaptive immunity, pro-inflammatory signaling, and anti-inflammatory signaling [13]. Chemokines are a subpopulation of cytokines that initiate the recruitment of well-defined leukocyte subsets through chemical stimuli [14]. Cells attracted to the chemokine follow a signal of increasing concentration towards the source of the chemokine, usually an infected or damaged cell. As cell signaling molecules, cytokines bind to receptors on the plasma membrane and elicit effects through the activation of an intracellular signaling cascade. Cytokines can be further classified based on the distance between the cell secreting the signaling ligand and the cell receiving the chemical signal [10]. Endocrine action is when cytokines pass through the bloodstream before reaching the target. Cytokines that act near the secreting cell are paracrine. Autocrine action is when cells can secrete a signal that is received through its own receptors. Cytokines have been characterized as belonging to one of six groups based on cytokine and cytokine receptor structure, but members may exhibit diverse functionality. The cytokine families and some of the members of each group are detailed in Table 1 [10].

**Table 1:** Details of six cytokine families and examples of family members.

Cytokine Family	Examples	Characteristics
Interleukin 1 family	IL-1α, IL-β, IL-RA	Induce responses against infection that are primarily pro-inflammatory IL-RA antagonizes effects of IL-1α and IL-β
Hematopoietin (Class I cytokine) family	IL-2, IL-4, IL-5, IL-6, IL-7, IL-9, IL-12p40, IL-12p70, IL-13, IL-15, IL-23, GM-CSF, G-CSF	Largest family of cytokines, all members sharing a 4-helix bundle structure Sequence and functional diversity Diverse effects include proliferation, differentiation, and antibody secretion
Interferon (Class II cytokine) family	IFN-γ, IL-10	Mediate early antiviral responses Activate macrophages, interact with cells of the adaptive immune system, and support the generation of Th1 cells
Tumor necrosis factor family	TNF-α, TNF-β1	Expressed in either soluble or membrane-bound form Cause apoptosis
Interleukin 17 family	IL-17	Primarily pro-inflammatory
Chemokine family	IL-8, Eotaxin, IP-10 (CXCL10), MCP-1(CCL2), MIP-1α (CCL3), MIP-1β (CCL4), RANTES (CCL5)	Secondary pro-inflammatory mediators Stimulate recruitment of well-defined leukocyte subsets Promote chemoattraction, movement of immune system cells into, within, and out of lymphoid organs

CCL, chemokine (C-C motif) ligand; CXCL, chemokine (C-X-C) ligand; G-CSF, granulocyte colony-stimulating factor; GM-CSF, granulocyte macrophage colony-stimulating factor; IFN-γ, interferon-γ; IL, interleukin; IL.1RA, IL-1 receptor antagonist; IP-10, IFNγ-inducible protein 10; MCP-1, monocyte chemotactic protein-1; MIP-1α, macrophage inflammatory protein-1α; MIP-1β, macrophage inflammatory protein-1β; RANTES, regulated on activation, normal T cell expressed and secreted; Th, T helper; TNF-α, tumor necrosis factor-α.

T and B lymphocytes mediate adaptive immunity. However, it is the T helper cells that are required for almost all adaptive immune responses [15]. They help activate B cells to secrete antibodies and macrophages to eliminate pathogens. Naïve T helper cells are differentiated into functional types defined by their pattern of cytokine production and function: Type 1 T helper (Th1), Type 2 T helper (Th2), T-regulator, and Th17 cells [16]. Proliferation and differentiation are functions of the particular cytokine milieu and signaling requirements during T cell receptor activation. Differentiation into either Th1 or Th2 effector cells then determines the nature of the subsequent adaptive immune responses activated by effector cells [17]. Th1 cells produce interferon (IFN)-γ, their signature cytokine and interleukin (IL)-2, while many also produce tumor necrosis factor (TNF)-α. Th2 signature cytokines are IL-4, IL-5, and IL-13, but these cells also make TNF-α, and some produce IL-9 and modest amounts of IL-2 [16]. Immune regulation is thought to require homeostasis between Th1 and Th2 activity [15]. If either Th1 or Th2 dominates, then the other response may be suppressed. Lower proportions of Th1 cells and higher proportions of Th2 cells have been found in children with ASD compared to healthy controls, providing evidence of an imbalance of Th1- and Th2-like cytokines in ASD [18]. In addition, analysis of peripheral blood mononuclear cells from children with ASD showed increased activation of both the Th1 and Th2 arms of the adaptive immune response, with a Th2 dominance and no compensatory increase in expression of the regulatory cytokine IL-10 [19].

Each cytokine can be produced by a single cell type or multiple cell types. For example, Th1 cells produce IFN-γ, IL-2, and TNF-β, while Th2 cells produce IL-4, IL-5, IL-6, IL-9, and IL-10 [20]. However, granulocyte macrophage colony-stimulating factor (GM-CSF) can be produced by multiple cell types, including macrophages, endothelial cells, and fibroblasts [21]. Similarly, IFN-β can be produced by multiple cell types including fibroblasts and epithelial cells. Cytokines can act on a single or multiple cell types. For example, IL-12 acts on Th1 cells, while IL-1 acts on T cells, B cells, macrophages, endothelial cells, fibroblasts, and epithelial cells, and all interferons act on multiple cell types. Cytokines also exhibit redundancy, meaning multiple cytokines exert the same biological action. Furthermore, an individual cytokine can have multiple effects on a target cell. IFN-γ induces antiviral proteins, upregulates major histocompatibility complex Class I antigens, stimulates NK and IL-12 production, and induces antiproliferative effects [22]. Increased levels of IFN-γ and IL-12 can induce inflammation whereas increased levels of transforming growth factor (TGF)-β, IL-4, and IL-10 can downregulate inflammation [23]. The classification of the biological action of cytokines as either pro-inflammatory or anti-inflammatory may be dependent on the amount of cytokine, the nature of the target cell, the nature of the activating signal, the nature of produced cytokines, and the timing and sequence of cytokine action [24].

## The Immune System, the Nervous System, Behavior, and the Role of Cytokines

The immune system and the nervous system are intricately interconnected. The functional status of the immune system affects a multitude of biological processes, including brain function and development, which can be affected when the innate and adaptive immune responses are dysregulated [25]. Sickness behavior, a term used to describe changes in the subjective experience and behavior occurring in a physically ill person [26], provides an example of how, through multiple mechanisms, the immune system can influence brain function and subsequent behavior [25]. Nonspecific symptoms of sickness behavior include fever, nausea, reduced appetite, fatigue, irritability, and withdrawal from physical and social environments [27]. Sickness behavior is considered an organized and evolved strategy to facilitate the role of fever in fighting infection. It is initiated by pro-inflammatory cytokines that are produced at the site of infection by activated accessory immune cells and is characterized by endocrine, autonomic, and behavioral changes [27]. The brain recognizes cytokines such as the pro-inflammatory cytokines IL-1α, IL-1β, TNF-α, and IL-6 as molecular signals of sickness [28]. Furthermore, TNF-α, IL-6, and IL-1β can cross the blood-brain barrier and act on the hypothalamus where they promote fever and sickness behavior [29]. The similarities of symptom expression in sickness behavior and depression have led to the hypothesis that cytokines and inflammatory factors are involved in the pathophysiology of neuropsychiatric disorders, and this has been a catalyst for extensive research into the pathways and mechanisms through which the immune system influences the brain and behavior [30]. Interestingly, another example of a relationship between symptom and cytokine expression involves immunotherapy in cancer patients, in whom prolonged exposure to the proinflammatory cytokine IL-2 results in dose- and time-related cognitive dysfunction and altered behavior [31].

The literature identifying abnormal cytokine profiles in depression, bipolar disorder, and schizophrenia [32–34] collectively suggests that cytokines induce both sickness behavior and neuropsychiatric symptoms, and that inflammation is a key pivotal factor in psychopathology [35]. In addition, a systematic review evaluating pro-inflammatory markers in almost 4,000 children and adolescents with neuropsychiatric and neurodevelopmental disorders, including ASD, identified preliminary evidence of the role of inflammation in these conditions and an association with a pro-inflammatory state [36]. Furthermore, growth in research into the role of inflammation has led to the redefinition of many diseases, such as heart disease, Alzheimer’s disease, type 1 diabetes, type 2 diabetes, and obesity, as inflammatory disorders [13]. A possible mechanism for the role of inflammation in these disorders is the alteration of the structural and functional integrity of the central nervous system (CNS) by cytokines, thereby contributing to the pathology of neuro-inflammation and neuropsychological disorders.

Peripheral cytokine signals are thought to access the brain through three pathways: humoral (with antibody involvement), neural, and cellular [30]. These communication pathways involve at least five mechanisms: (1) passage of cytokines through leaky regions of the blood-brain barrier; (2) active transport *via* saturable cytokine-specific transport molecules on brain endothelium; (3) activation of endothelial cells, which release second messengers within the brain parenchyma; (4) transmission of cytokine signals *via* afferent nerve fibers, including the vagus; and (5) entry into the brain parenchyma of peripherally-activated monocytes which release cytokines.

Cytokines may influence behavior through effects on neurotransmitter function, neuroendocrine activity, neurogenesis, and alterations to brain circuitry [30]. For example, cytokines have been shown to increase the release and decrease reuptake of the excitatory neurotransmitter glutamate, which can result in the pathological process of excitotoxicity [37]. This type of mechanism could support a model for some types of ASD; the model postulates an increased excitation/inhibition ratio in key neural systems, such as sensory, mnemonic, social, and emotional systems [38].

An alternate communication pathway has recently been proposed based on the groundbreaking work by Louveau and colleagues who identified functional lymphatic vessels in the CNS that carry fluid and immune cells from the cerebrospinal fluid, and in doing so discovered a pathway for immune cells to exit the CNS [39]. While the anatomy and functional importance of these pathways and systems have yet to be characterized in humans, this work provides a new perspective on the possible etiology of neuroinflammatory and neurodegenerative conditions. These findings also provide an impetus for further consideration of the relationship between immune responses and behaviors in other conditions that are characterized by immune system dysfunction, such as ASD.

An understanding of the pathways and mechanisms through which the immune system affects behavior is primarily based on findings in animal models. For example, mice deficient in T cells have cognitive deficits [25]. Interestingly, an altered activation profile for T cells has been identified in ASD, with these perturbations of T cell function possibly modulating behavior and core features of ASD [40]. Recently, social behavior as operationalized within an animal model, has been shown to be influenced by meningeal immunity [41]. Mice deficient in adaptive immunity, specifically an absence of interferon IFN-γ, display both hyper-connectivity in the prefrontal cortex (PFC) and significant social deficits. This is particularly interesting, given that hyperactivity in the PFC in the context of social stimuli is known to be a feature of social impairment in ASD [42]. Filiano and colleagues demonstrated that CNS neurons respond to IFN-γ derived from meningeal T cells, elevating tonic GABAergic inhibition [41]. This process prevents aberrant hyper-excitability in the PFC and restores social behaviors through IFN-γ. Previously, IFN-γ released from T cells was thought to predominantly stimulate and modulate immune responses to infection. Interestingly, NK cells, recognized as major producers of cytokines including IFN-γ [12] in physiological and pathological conditions, are dysfunctional in ASD [43]. While a novel finding, the regulation of neural activity and social behavior through IFN-γ provides further evidence for the interconnectedness of the immune system, the nervous system, and behavior.

## Immune System Deregulation in ASD

Evidence suggesting a pathophysiological relationship between the immune system and ASD was first presented over 40 years ago [44]. Subsequent research investigating the complex relationship between the immune system and ASD symptomatology has identified numerous potential interactions and proposed associated mechanisms at both the systemic and cellular levels [7, 45]. One of these areas of research focuses on the prenatal period. Maternal immune activation refers to the defensive response of the mother’s immune system to an invading pathogen. A large population-based study found that acute immune activation caused by maternal viral infection during the first trimester increases the risk of ASD in children [46]. Furthermore, a recent meta-analysis of >40,000 ASD cases showed that maternal infection during pregnancy is associated with an increased risk of ASD in the offspring, with hospitalization during infection heightening the risk [47]. Moderators of this risk include the type of infectious agent, the timing of infectious exposure, and the site of infection. A recent review has proposed that maternal infection leads to the release of pro-inflammatory cytokines and activation of Th17 cells in the mother’s bloodstream and that the immune status and genetic predisposition of the fetus determine its vulnerability to maternal immune activation, a process considered a disease primer [48]. Peripheral cytokine profiles at birth, including elevated IL-1β and IL-4, are associated with an ASD diagnosis later in childhood and vary with ASD symptom severity [49]. Elevation of IL-1β and IL-4 may reflect a prenatal immune challenge, and an association with both ASD risk and cognitive developmental outcomes suggests the possibility of a global impact of early cytokine dysregulation [49]. Familial autoimmunity has also been implicated in the pathogenesis of ASD, with an increased risk of ASD in children with a maternal history of rheumatoid arthritis and celiac disease, and an increased risk of infantile autism has been identified in children with a family history of type 1 diabetes [50].

Another area of focus on immune involvement in the pathogenesis and maintenance of ASD is the postnatal period. Altered cytokine profiles have been consistently linked to ASD in children during this period [7]. In high-functioning male children with ASD, the plasma levels of IL-1β, IL-1 receptor antagonist (IL-1RA), IL-5, IL-8, IL-12(p70), IL-13, and IL-17 are elevated relative to matched controls [51]. IL-1β, a pro-inflammatory cytokine, activates neutrophils and macrophages to phagocytose invading pathogens [52]. IL-1RA inhibits the activities of IL-1β, suggesting that the levels of IL-1RA might be a function of a negative feedback regulator role in response to the elevation of IL-1β [51]. IL-1β is involved in the production of IL-17 [53] and IL-17 is a potent mediator of the production of IL-8, a chemokine with important roles in the innate immune response. IL-5 and IL-13 stimulate B cells to secrete immunoglobulins including IgE, which is a mediator of allergic inflammation. IL-12(p70) is a pro-inflammatory cytokine that enhances Th1 and NK cell responses [54]. In addition to elevated expression of IL-1β, as identified by Suzuki and colleagues, IL-6, IL-12, TNF-α, and IL-23 are also elevated in ASD compared to healthy controls, suggesting a dysregulated immune response [55]. TNF-α is a central regulator of inflammation and is elevated in the cerebrospinal fluid of children with ASD [56]. IL-6 is typically regarded as a pro-inflammatory cytokine and has been identified as a cytokine the brain recognizes as a molecular signal of sickness [28]. However, it also has regenerative or anti-inflammatory activity, and is involved in the regulation of metabolic and neural processes [57]. Finally, a further example of immune abnormalities in the postnatal period involves activation of the monocytic and Th1 arm of the immune response, *via* increased IL-1RA and increased IFN-γ, respectively, and this has been found in children with ASD [58].

Immune-mediated mechanisms have also been hypothesized as reflecting a chronic state of specific cytokine activation [59]. Immunocytochemical studies have identified marked activation of microglia and astroglia associated with the increased production of two cytokines by neuroglia, macrophage chemoattractant protein (MCP)-1, and TGF-β1 [59]. In addition, a unique profile of pro-inflammatory cytokines has been identified in cerebrospinal fluid [59]. Another post-mortem study also demonstrated significant increases in pro-inflammatory and Th1 cytokines relative to matched controls [60]. Elevation of IL-6 in ASD, both centrally and peripherally, has been frequently reported [59–62]. In a mouse model with elevated IL-6 in the brain, Wei and colleagues have shown that IL-6 can modulate autism-like behaviors through impairments of synapse formation, dendritic spine development, and neuronal circuit balance [63]. Acute and chronic psychological stress and alterations in sleep duration and quality, a commonly reported comorbidity in ASD [64], increase the concentrations of IL-6 [65]. This evidence for abnormal cytokine profiles in ASD suggests that immune system disturbances may be active and continuous contributors to the presentation of ASD. It is this accumulation of evidence that has acted as the catalyst for efforts to characterize possible subgroups of ASD patients who present with immune system abnormalities or dysfunction and altered patterns of symptom presentation [9, 66].

Associations between changes in peripheral cytokine expression and the severity of behavioral impairments and associated symptoms have been identified in children with ASD (Table 2). Reduced levels of the regulatory cytokine TGF-β1 are associated with reduced adaptive behavior and worsening behavioral symptoms [67, 68]. While TGF-β1 is involved in cell growth and differentiation, organ development, migration, and apoptosis, its major role is to control inflammation. This negative correlation with behavioral impairment suggests that there is an ongoing inflammatory process in children with ASD who present with worsening behavioral profiles [67]. Increased levels of the chemokines MCP-1, RANTES (regulated on activation, normal T cell expressed and secreted), and eotaxin are associated with more impaired behaviors and adaptive functioning [69]. Chemokines are expressed in the developing brain and regulate neuronal cell migration, proliferation, and differentiation. They are also involved in communication between neurons and microglia [70]. Elevated IL-1β and IL-6 have been associated with increased stereotypical behaviors [62]. Dysregulation of IL-1β, a pro-inflammatory cytokine expressed early in an immune response, is implicated in impairments in memory and learning [71]. IL-1β induces and inhibits neural progenitor cell proliferation in the CNS, which can contribute to region-specific deviant brain growth in ASD [72]. As previously highlighted, IL-6 is elevated in most inflammatory states and has been implicated in a wide range of conditions. Elevation of IL-8 and IL-12p40 is also associated with greater impairment of aberrant behaviors including lethargy and stereotypy as measured by the Aberrant Behavior Checklist (ABC) [62]. In addition, as the expression of IL-8 decreases, cognitive and adaptive ability improves [62]. IL-8 is a chemoattractant cytokine, attracting and activating neutrophils in regions of inflammation [73] and hence may contribute to the pathogenesis of inflammatory diseases [74].

**Table 2:** Cytokines and associations with severity of autism-related symptoms.

Cytokine	*n* (ASD)	Medium	Measures	Finding	Study
TGF-β1	75	Plasma	ABC, VABS	Reduced levels associated with reduced adaptive behavior and worsened behavioral symptoms including stereotypy, irritability, and hyperactivity	Ashwood *et al.* [67]
Eotaxin	80	Plasma	ABC, MSEL, VABS	Increased levels associated with increased severity of lethargy, stereotypy, and hyperactivity (ABC)	Ashwood *et al.* [69]
				Increased levels associated with greater impairments in visual reception, fine motor skills, and receptive and expressive language (MSEL)	
				Increased levels associated with greater impairments in communication, daily living skills, socialization, and motor skills (VABS)	
MCP-1	80	Plasma	ABC, MSEL, VABS	Increased levels associated with greater impairments in visual reception, fine motor skills and expressive language (MSEL)	Ashwood *et al.* [69]
				Increased levels associated with greater impairments in daily living skills (VABS)	
RANTES	80	Plasma	ABC, MSEL, VABS	Increased levels associated with increased severity of lethargy, stereotypy, and hyperactivity (ABC)	Ashwood *et al.* [69]
				Increased levels associated with greater impairments in visual reception, fine motor skills, and expressive language (MSEL)	
				Increased levels associated with greater impairments in communication and socialization (VABS)	
IL-1β	97	Plasma	ADI-R, ABC, MSEL, VABS	Increased levels associated with increased stereotypy (ABC)	Ashwood *et al.* [62]
IL-4	97	Plasma	ADI-R, ABC, MSEL, VABS	Increased levels associated with greater impairments in non-verbal communication (ADI-R)	Ashwood *et al.* [62]
IL-6	97	Plasma	ADI-R, ABC, MSEL, VABS	Increased levels associated with increased stereotypy (ABC)	Ashwood *et al.* [62]
IL-8	97	Plasma	ADI-R, ABC, MSEL, VABS	Increased levels associated with increased hyperactivity, stereotypy, and lethargy (ABC)	Ashwood *et al.* [62]
				Decreased levels associated with improved visual reception, receptive language, and expressive language (MSEL)	
				Decreased levels associated with improved daily living skills (VABS)	
IL-12p40	97	Plasma	ADI-R, ABC, MSEL, VABS	Increased levels associated with increased stereotypy and lethargy (ABC)	Ashwood *et al.* [62]
TGF-β1	30	Plasma	CARS	Reduced levels associated with increasing severity	El Gohary *et al.* [68]

ABC, Aberrant Behavior Checklist; ADI-R, Autism Diagnostic Interview—Revised; IL, interleukin; MCP-1, monocyte chemotactic protein-1; MSEL, Mullen Scales of Early Learning; RANTES, regulated on activation, normal T cell expressed and secreted; TGF-β1, transforming growth factor-β1; VABS, Vineland Adaptive Behavior Scales; WAIS-R, Wescheler Adult Intelligence Scale-Revised.

Other cellular markers of immune dysfunction identified in children with ASD include significantly higher absolute numbers of B cells and NK cells, and increased markers of cellular activation compared to healthy controls [75]. These findings suggest an immune response activation that leads to an increased frequency of NK cells and activated B cells and T cells. Increased levels of the IgG4 subclass have been identified in children with ASD [76]. The IgG4 subclass has features and biological function different from other subclasses of IgG, acting as a blocking antibody that binds strongly to antibody receptors rather than a protective antibody. A correlation between the severity of behavioral measures and reduced levels of immunoglobulin has also been found, suggesting suboptimal humoral function in children with ASD [77]. Furthermore, an elevated prevalence of other immune-related comorbidities, including autoimmune diseases, allergies, and psoriasis, has been found in children with ASD compared to healthy controls [6]. Overall, these relationships between immune dysfunction and behavioral symptoms associated with an ASD presentation suggest an ongoing relationship impacting the severity of the condition in children with an ASD diagnosis.

## Altered Cytokine Profiles as Potential Biomarkers in ASD

To facilitate improved communication about measurements of disease and treatment effects, an expert working group convened by the National Institutes of Health Director’s Initiative on Biomarkers and Surrogate Endpoints (USA) proposed the following definition of a biological marker or biomarker: a characteristic that is objectively measured and evaluated as an indicator of normal biological processes, pathogenic processes, or pharmacologic responses to a therapeutic intervention [78]. Additional applications of biomarkers have been proposed, such as use as a diagnostic tool for the identification of those patients with a disease or abnormal condition, and for the prediction and monitoring of the clinical response to an intervention. Specifically, with respect to using biomarkers to guide better treatment of schizophrenia and other psychotic disorders, Banati and Hickie have proposed clinically useful properties of biomarkers, including diagnostically non-specific, quantitative, longitudinal, plausibly linked to underlying pathophysiology, and predictive of risk of impairment [79]. They also highlight the clinical importance of the role of biomarkers in guiding treatment selection, and demonstrating a correlation between active interventions and the short-term clinical response.

Numerous biomarkers have been proposed for ASD, including biochemical, morphological, immunological, hormonal, neurophysiological, neuroanatomical, and neuropsychological markers [80]. A recent clinical trial in ASD involving an immunomodulatory pharmacological intervention demonstrates a correlation between an active intervention and a relevant short-term clinical response. Greater improvement in symptoms of irritability, hyperactivity, stereotypic behavior, social withdrawal, and inappropriate speech was achieved when risperidone was used adjunctively with pentoxifylline, an immune-modulating drug and pro-inflammatory cytokine inhibitor [81]. Furthermore, another adjunct treatment trial in children with ASD using risperidone and pioglitazone showed reductions in the severity of symptoms of irritability, social withdrawal, and hyperactivity in the adjunct treatment group compared to the risperidone-only group, indicating positive effects of pioglitazone [82]. Pioglitazone is a peroxisome proliferator-activated receptor, which inhibits the production of pro-inflammatory cytokines and chemokines by microglia [83, 84]. Furthermore, risperidone was found to be more effective when given adjunctively with celecoxib, a nonsteroidal anti-inflammatory drug, with significant improvements in the irritability, social withdrawal, and stereotypy subscales of the ABC [85]. However, in a recent open-label study of risperidone treatment for children and adolescents with ASD, the plasma levels of eotaxin and MCP-1 showed statistically significant decreases after treatment, although these changes were not significantly associated with changes in severity measures [86]. Eotaxin and MCP-1 are pro-inflammatory and are elevated in brain specimens from patients with ASD [59]. Overall, the results of these trials suggest that treating immune-related symptoms may contribute to behavioral changes in ASD.

The clinical trials of immune-modulating agents, taken together with previous examples of immune system perturbations in children with ASD, suggest that cytokines are worthy of consideration as potential biomarkers of a subgroup of individuals with an ASD diagnosis and more severe behavioral outcomes. However, the validity of peripheral sampling of blood cells as a relevant biomarker and as a surrogate for a CNS sample is still debated. Given that peripheral blood cells comprise the major cellular components of the immune system, they could be considered suitable for the assessment of immune-related markers [87]. In addition, accessibility and speed of sampling for regular assessments and analysis of peripheral blood sampling are significant advantages over, for example, cerebrospinal fluid sampling and brain imaging.

A National Research Council (USA) report on precision medicine, which is the tailoring of medical treatment to the individual characteristics of each patient, highlights a critical need for the deconstruction of current diagnostic groups using biomarkers to help identify the subgroups for which treatment is highly effective [88, 89]. In addition to physical signs and symptoms, it is recommended that conditions should also be defined by their underlying molecular causes and other factors, which would represent an emergence of a new taxonomy based on biomedical research and an extensive patient data network. A data network would be needed to integrate current research on the molecular composition of conditions with clinical data on individual patients in an effort to drive precision medicine. Precision medicine also refers to the classification of individuals into subpopulations that differ in their susceptibility to a particular condition in the biology and/or prognosis of those conditions they may develop, or in their response to a specific treatment [89]. Similarly, the long-term aim of the Research Domain Criteria (RDoC) project of the National Institute of Mental Health (USA) is precision medicine for psychiatry through the adoption of a diagnostic system based on a comprehensive understanding of the biological and psychosocial bases of conditions, unhindered by the limitations of diagnostic categories [88].

A robust biological system is one that maintains its state and functions against external and internal perturbations [90]. It is possible that a robust immune system is protective and that while the mechanisms leading to abnormal function have yet to be established, it is tempting to consider that a regulated and functional immune system, both pre- and postnatally, is a prerequisite for a normal functioning brain [91]. Consistent with the RDoC agenda, the non-specific association found between inflammation and neuropsychiatric disorders, including ASD, and the identification of common reliable inflammatory markers across those different disorders warrant further investigations to determine if these processes play a role in the etiology of symptom dimensions or symptom domains that overlap or are shared by different conditions [35]. A diagnosis of ASD continues to be behaviorally defined. However, the body of research and accumulating evidence with respect to immune system perturbations in ASD suggest that a broader approach should be taken in order to understand biological systems as they pertain to ASD and associated behaviors.

## Conclusion

The hallmark heterogeneity of ASD is a key reason for the focus of researchers on the identification of potential biological measures as a means of describing subsets within ASD, and thereby facilitating the targeting of more individualized therapies. Previously discussed cytokine aberrations in ASD have highlighted a possible relationship between cytokine aberration and ASD. Altered cytokine levels may facilitate the identification of ASD subtypes that share similar traits and profiles, as well as provide biological markers that facilitate monitoring of the benefits of active treatments over the time-course of clinical trials. Biological markers, as objective measures of the response to treatment in clinical trials, will assist with the identification of efficacious interventions. Furthermore, the identification of objective markers of a pathological state related to a subgroup in ASDs may assist in reducing the heterogeneity of participants in clinical trials, and this may lead to the identification of more targeted treatments for autism-related symptoms.
